# Learning from Power Signals: An Automated Approach to Electrical Disturbance Identification within a Power Transmission System

**DOI:** 10.3390/s24020483

**Published:** 2024-01-12

**Authors:** Jonathan D. Boyd, Joshua H. Tyler, Anthony M. Murphy, Donald R. Reising

**Affiliations:** 1 Tennessee Valley Authority, Chattanooga, TN 37402, USA; jboyd7@tva.gov (J.D.B.); ammurphy@tva.gov (A.M.M.); 2 Electrical Engineering Department, The University of Tennessee at Chattanooga, Chattanooga, TN 37403, USA; joshua-tyler@mocs.utc.edu

**Keywords:** Digital Fault Recorder (DFR), Power Quality (PQ), Electrical Disturbance, identification, machine learning

## Abstract

As power quality becomes a higher priority in the electric utility industry, the amount of disturbance event data continues to grow. Utilities do not have the required personnel to analyze each event by hand. This work presents an automated approach for analyzing power quality events recorded by digital fault recorders and power quality monitors operating within a power transmission system. The automated approach leverages rule-based analytics to examine the time and frequency domain characteristics of the voltage and current signals. Customizable thresholds are set to categorize each disturbance event. The events analyzed within this work include various faults, motor starting, and incipient instrument transformer failure. Analytics for fourteen different event types have been developed. The analytics were tested on 160 signal files and yielded an average accuracy of 99%. Continuous nominal signal data analysis was performed using an approach called the cyclic histogram. The cyclic histogram process is intended to be integrated into the digital fault recorders themselves in order to facilitate the detection of subtle signal variations that are too small to trigger a disturbance event and that can occur over hours or days. In addition to reducing memory requirements by a factor of 320, it is anticipated that cyclic histogram processing will aid in identifying incipient events and identifiers. This project is expected to save engineers time by automating the classification of disturbance events and increasing the reliability of the transmission system by providing near real-time detection and identification of disturbances as well as prevention of problems before they occur.

## 1. Introduction

The continued and increasing deployment of “smart” devices (e.g., switches, relays, etc.) within power utility generation, transmission, and distribution infrastructure has led to an ever-growing amount of event data being recorded and stored. Processing and analysis of such event data have traditionally been conducted by power utility personnel using “by-hand” approaches. By-hand approaches rely heavily upon the knowledge, experience, and expertise of the person or persons conducting the analysis, and severely limit the number of events that can be analyzed within a given period. These limitations are exacerbated considering that: (i) power utilities cannot dedicate personnel solely to event processing and analysis, and (ii) the analysis is often conducted hours if not days after the event, making it of limited value. This raises the question of whether an automated approach can be developed to analyze events in order to provide actionable information to power utility personnel. The present work aims to answer this question by creating an automatic rule-based process that leverages institutional knowledge to identify events based on the signatures and features they impart to the voltage and current waveforms.

The recent emergence of Deep Learning (DL) has led to its application in the classification of power system events. A multi-layer Deep Neural Network (DNN) was used by the authors of [1] to classify power events using statistics calculated from voltages and frequencies. In [2], the authors used a DNN to detect short circuits and earth faults. The authors of [3] used a Convolutional Neural Network (CNN) to classify overlapping and successive events. A CNN and Artificial Neural Network (ANN) were used by the authors of [4] to classify outage events collected in an operational power distribution system. A CNN was used to classify Power Quality (PQ) events in synchro-waveform measurements made in a power distribution system in [5], while a Long Short-Term Memory (LSTM) classifier was used in [6] and a Graph CNN (G-CNN) in [7]. However, these works require a sufficiently large set of labeled training data. To overcome this requirement, the authors of [8,9] used semi-supervised DL algorithms, for which only a minimal set of labeled training data is required. The use of DL-based classification is advantageous because these algorithms have been shown capable of achieving high accuracy levels; however, this is at the expense of requiring large datasets for training [10]. Choosing, setting, or tuning of multiple settings, parameters, and hyperparameters can be complex, to the extent of requiring the use of a separate algorithm or a grid search to achieve optimal results. DL-based processes lack explainability, which can inhibit use by power utility personnel due to a lack of clarity on how decisions are reached [11]. Moreover, forgetfulness that can occur when training a DL algorithm to learn new information. One scenario in which this is problematic is in cases where new classes need to be added, which happens when sufficient training data have been collected within the power system.

In [12], the authors detailed a rule-based approach for categorizing PQ events using the S Transform (ST). The data used in this approach are a mix of simulated data and real-world data from the power system. The Fourier Transform (FT) and Short-Time Fourier Transform (STFT) cannot effectively extract each signal’s unique features, while the Wavelet Transform (WT) has been used to extract time and frequency domain characteristics simultaneously, it is somewhat vulnerable to noise, and is computationally expensive. The ST can be considered a hybrid between the STFT and WT, as it has the time and frequency domain characteristics while using a variable window length to provide information at different resolutions. The ST has been shown to provide higher noise immunity. Finally, categorization of the PQ events was performed using Artificial Neural Networks (ANNs), fuzzy logic, decision trees, and others. The ST contours highlight the distinctive features present within the original PQ event signal, such as voltage sags. A set of rules is then defined to set the thresholds to trigger certain event types. These rules rely heavily upon the knowledge of PQ experts, and a dataset containing distorted signals is used to determine the corresponding threshold values. The rules are designed to separate the events into three categories: magnitude disturbances, transients, and signal distortion. The tests performed on the signals include positive and negative tests for an extra classification layer. This approach is very portable to other applications due to the normalization of the voltage, which facilitates the usage of any voltage level. The results from [12] heavily favor the rule-based ST approach. This approach was able to classify disturbances with 98% accuracy, while a traditional ANN method achieved an accuracy of 92%. The rule-based method can withstand considerable noise in the signal; one reason for this superior accuracy is that the rule-based approach is more specialized against each type of disturbance than the ANN approach.

The approach presented in [13] used a machine learning approach augmented by including the Kullback–Leibler (KL) divergence measure and standard deviation. The KL divergence is very efficient, as it can be applied to a single signal cycle. The KL divergence calculates the probability of a particular cycle being a member of two or more events. The standard deviation is used, as it is very effective in detecting PQ disturbances. These two methods are used for each cycle of the disturbed signal and compared with an ideal sinusoidal signal to capture the disturbance. After the detection phase, the classification phase is performed using a Support Vector Machine (SVM) to determine a decision boundary between event types. This method proved very effective in differentiating between voltage sag and swell events. However, because voltage flicker and swell are more similar than sag and swell, this approach is unlikely to function as effectively. Overall, this method achieved an accuracy of 94.02%.

The approach presented in [14] provides a novel PQ disturbance classification method. The method extracts features from the cross-correlogram of the PQ disturbances. The positive and two adjacent negative peaks are the classification features. These three values are then fed into a fuzzy-based classification system. One drawback to the approach in [14] is its use of simulated data, which means that the classification accuracy may change when real-world data are used. The two types of correlation are cross-correlation and auto-correlation. Cross-correlation measures the strength of similarity between two signals, while auto-correlation measures the cross-correlation of a signal with itself. To detect disturbances, [14] calculates the cross-correlation response between an ideal signal and a disturbed one. A fuzzy logic classifier allows for uncertainty in a logic system. Because human experts design the rules in the fuzzy system, the system is only as good as those who created it. The system used in this approach was a Mamdani-type inference system with three inputs and one output. Eighteen linguistic variables were used for the output membership function to determine the PQ event classification. This classifier was tested using seventy generated signals and achieved an accuracy of 100%. The accuracy remained 100% even when noise was added to the test signals.

The work presented herein uses algorithms developed in a programming platform that classify various PQ events into one or more categories. The developed algorithms are rule-based with customizable thresholds based on engineers’ expertise. Each PQ event’s signal data are stored in a Comma Separated Values (CSV) file generated by the field device containing a time vector, three voltage phases, and three current phases. An executable file is initiated to read each CSV file into a working directory and then categorize them as particular PQ event type(s). This accounts for the case of multiple PQ event types occurring and being recorded within the same CSV file. A CSV file is then generated with the classification results and analytic outputs, such as the magnitude of the current. There are several differentiating factors that make the presented work unique and preferable to other methods:

- The proposed automated process was developed and tested using real-world data rather than simulated data. All data were recorded by smart field devices—PQ monitors and Digital Fault Recorders (DFRs)—operating in a high-voltage transmission system.
- The rule-based method employs a tailored approach that exploits specific characteristics of each PQ event, thereby eliminating the need for large training datasets. This makes it ideally suited to real-world settings and deployments, where certain PQ events occur very rarely, i.e., data availability dictates our approach).
- Rule-based methods mimic the expertise of engineers in order to ease interpretation and understanding of the classification results by power system personnel, thereby overcoming the explainability and forgetfulness issues associated with DL and other Artificial Intelligence (AI)-based approaches.
- The developed process uses very few functions specific to any programming platform. This reduces the need for expensive licenses while allowing the algorithms to be translated into other programming languages and software based on the specific needs of the power utility. This approach is adopted to facilitate widespread use of the developed algorithms across the power industry.
- The rule-based nature of the developed process allows every threshold to be changed as needed by power utility personnel based on performance or system specifics. This paper defines empirical thresholds as τ in equations and using **bold** lettering in the text.
- The methods used are very detailed; in addition to simple signal characteristics such as voltage sag and swell, they can predict specific disturbances occurring in the power system (e.g., ferroresonance).
- Because its rules are developed on a per-event basis, the rule-based process allows new events to be added without requiring changes to those already integrated. In contrast, a DL-based approach requires retraining the entire model to add a new event, as the DL algorithm decision is based on learned relationships that depend on all events, not just the new one.
- The rule-based approach can assign unrecognized events to an “unknown” category, thereby mitigating misclassification errors. In contrast, DL-based approaches must assign all inputs to one of the model’s known classes, as they typically lack an “unknown” class.

Another aspect of the project was to analyze continuous oscillography data stored on the DFRs. Each day of data can be as much as 20 to 50 gigabytes (GB), which is far too much data for an engineer to analyze manually. Due to onboard memory constraints, each DFR stores two weeks of continuous oscillography data before it is overwritten. The approach presented in this work uses a method known as a cyclic histogram [15] to reduce an average day’s 35 GB worth of continuous oscillography data to 72 megabytes (MB). This memory reduction increases the time window during which the data can be stored from two to roughly one thousand weeks while allowing engineers to monitor for trends and subtle deviations in continuous signal data that have not produced any disturbances large enough to trigger a DFR event.

The remainder of this paper is organised as follows. Section 2 presents the methodology, including general calculations, continuous waveform analysis, and the various disturbance event types. Section 3 provides the results of each event type and the continuous waveform analysis. Section 4 provides a summary and describes opportunities for future work.

## 2. Methodology

This section first presents descriptions of calculations, analyses, and tests used to categorize multiple events. A specific event may require the threshold of one or more of these general calculations, analyses, or tests to be changed and are detailed under the specific event being categorized. Next, the developed and employed methodologies for categorizing specific events and continuous signal processing using the cyclic histogram are described. The methodology is presented in this way because our approach allows electrical disturbances to be identified within a CSV file in parallel, series, or individually in order to maximize its flexibility and usability to power utility personnel and make its adoption and use easier.

### 2.1. General Calculations, Analyses, and Tests

This section describes the calculations, analyses, and tests used to identify more than one event.

#### 2.1.1. Calculating Nominal Values

The first task in processing a voltage or current signal is to calculate nominal values from the data. The sampling frequency is calculated by

(1) $$F_{s}=\frac{N}{t_{e}-t_{1}},$$

where $F_{s}$ is the sampling frequency in Hertz (Hz), *N* is the number of samples in the time vector, and $t_{1}$ and $t_{e}$ are the first and last values of the time vector, respectively. After the sampling frequency is known, the nominal number of samples in each cycle is determined by

(2) $$N_{c}=\frac{F_{s}}{F_{n}},$$

where $N_{c}$ represents the number of samples per cycle, $F_{s}$ is the sampling frequency, and $F_{n}$ is the nominal frequency of the power system, which is assumed to be 60 Hz.

Generally, PQ event records capture several voltage or current signal cycles before a disturbance begins. The DFRs recording the data used in this work are typically set to record fifteen cycles before a disturbance. The nominal peak values of voltage and current signals are determined using these “pre-event” cycles for each processed signal. For this work, the first cycle in the event record was used to determine these nominal scalar values, denoted as (i) ${\hat{V}}_{q}$ for nominal peak voltage, (ii) ${\hat{I}}_{q}$ for nominal peak current, (iii) ${\bar{V}}_{q}$ for nominal Root Mean Square (RMS) voltage, and (iv) ${\bar{I}}_{q}$ for nominal RMS current. The magnitudes of the voltage and current signals are compared to these nominal values in order to normalize the data to the power system’s particular voltage or current level, allowing for more flexibility when using these tools at different scales within the system.

#### 2.1.2. Root Mean Square

The RMS of a signal is another characteristic used to classify electrical disturbance events. A signal’s RMS is provided by

(3) $$\bar{x}=\sqrt{\frac{1}{N_{w}}\sum_{i=1}^{N_{w}}{\left|x[i]\right|}^{2}},$$

where *x* is the analog signal, $N_{w}$ is the size of the RMS window, and $\bar{x}$ is the RMS calculation of the analog signal [16]. Unless otherwise stated, we set the size of the RMS window to the nominal number of samples in each cycle, $N_{c}$.

One use of RMS is in determining whether the signal value is non-zero. In the instantaneous case, the sinusoidal signal crosses zero every half-cycle, making it more difficult to tell whether the value remains near zero. A signal’s RMS is used in events such as motor starting, where the current increases over time.

#### 2.1.3. Differentiation

One of the most common calculations is a signal’s derivative. Equation (4) represents the first derivative concerning the number of samples.

A positive first derivative indicates that the signal is increasing, while a negative first derivative suggests that the signal is decreasing. This fact is used to detect the presence of peaks or spikes within a signal. The maximum or minimum of a peak or spike corresponds to the first derivative changing sign, i.e., from positive to negative or vice versa. A change in the first derivative’s sign is calculated by

(4) $$x^{\prime }\left(n_{1}\right)\times x^{\prime }\left(n_{2}\right)<0$$

where $x^{\prime }$ is the first derivative of the analog signal, $n_{1}$ is the sample before the first derivative’s sign changes, and $n_{2}$ is the sample after the sign changes. Multiple sign changes over a short time interval provide a strong indication that a transient disturbance is present within the signal being processed.

**Figure 1:**
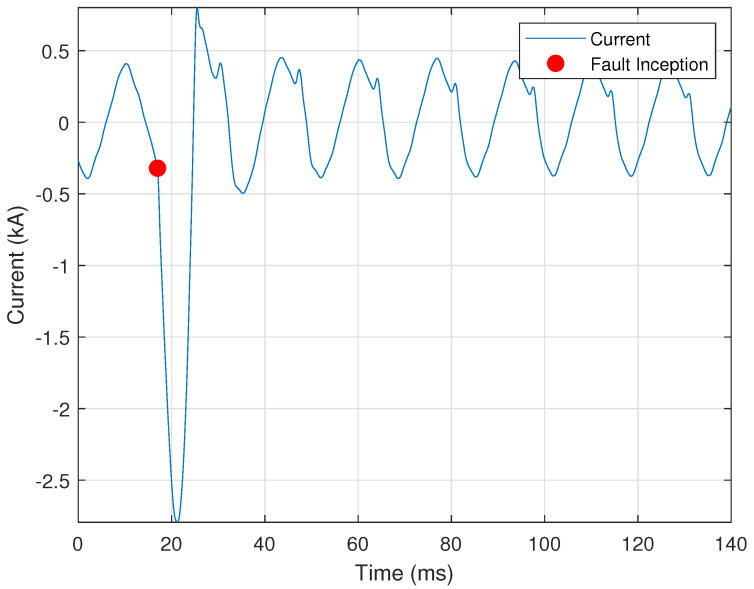
Fuse fault showing the use of the second derivative test.

The second derivative is used to determine the change in the slope of the curve. A sudden increase in the second derivative shows a sudden increase in slope, and can indicate the point at which a fault begins. Figure 1 provides a representative illustration showing the use of the second derivative in determining the start of a fuse fault. The red circle shown is where the second derivative is higher than an empirical threshold, indicating a sudden increase in the slope of the curve. The third derivative is used to detect a shift in the slope of a curve.

#### 2.1.4. Harmonic Ratios

Harmonics can be key indicators of particular events within a transmission system, e.g., current transformer saturation, harmonic resonance, etc. Harmonic analysis is facilitated by calculating the harmonic ratio, which is useful in determining the dominant frequency components within a signal. The *n*th harmonic ratio is calculated by

(5) $$H_{n}=\frac{|X_{n}|}{|X_{1}|},$$

where *X* is the Fast Fourier Transform (FFT) of *x*, $|X_{1}|$ is the magnitude of the fundamental frequency (i.e., 60 Hz), and $|X_{n}|$ is the magnitude of the *n*th multiple of the fundamental frequency [17].

#### 2.1.5. First Cycle Comparison

**Figure 2:**
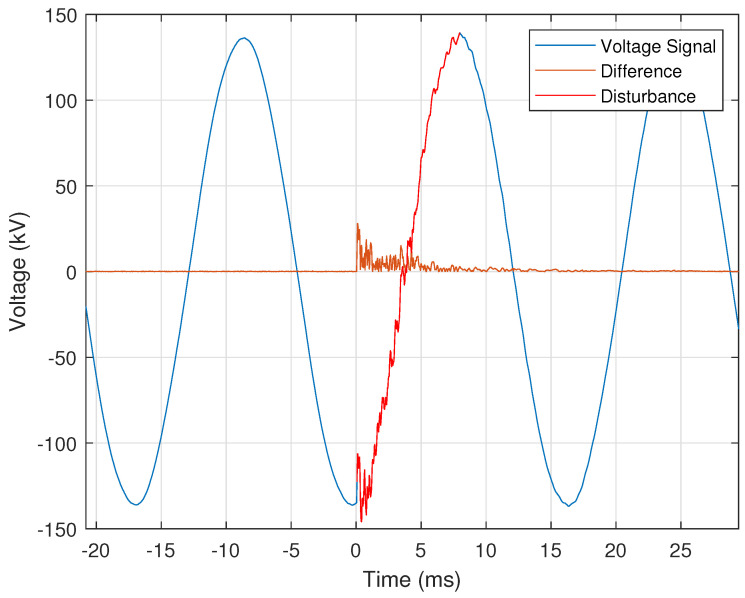
Voltage signal showing disturbance during capacitor switching.

The CSV files generally store at least fifteen cycles of the nominal voltage and current signals before the disturbance event; thus, a useful disturbance detection approach is to compare the signal’s first cycle with each remaining cycle within the CSV file. After the first cycle is selected, it is replicated to construct an ideal signal of the same length as the recorded signal from which the first cycle was extracted. The generated ideal signal is then subtracted from the recorded signal. The time indices at which this difference is very high indicate the start of a disturbance. Figure 2 illustrates the application of this approach in detecting the start of a capacitor switching event within a recorded voltage signal. Figure 2 shows the voltage signal, with the capacitor switching disturbance portion of the signal highlighted and the result of the difference calculation overlaid. The difference calculation is highest corresponds to the start of the capacitor switching event, which is assigned a start time of zero milliseconds.

### 2.2. Continuous Signal Processing

In addition to classifying disturbance events, this work uses cyclic histograms to reduce the memory storage requirements associated with using DFRs to continuously record signal data. This work extends the cyclic histogram concept by generating residual and frequency histograms. The cyclic histogram was first proposed in [15] to significantly reduce the size of continuously recorded oscillography data. This size reduction allows data to be stored much longer than the oscillography (OSG) file. PQ analysts can pull data from each DFR without straining the telecommunications network. A script was created to perform the following tasks:

- Read the most recent configuration (CFG) file and extract the necessary data to read and correctly interpret the matching OSG file.
- Time-synchronize each cycle reliably in order to generate the cyclic and residual histograms.
- Perform a custom “maximum frequency” calculation to generate a faster and less computationally intense frequency histogram than traditional FFT processing.
- Generate six CSV files; each of the three histogram types corresponds to a CSV containing the histogram along with an accompanying metadata file that stores the bin values and record dates.

Signals are analyzed based on a sine representation; thus, the continuous signal data are processed using a negative-to-positive transition in the cycle. This negative-to-positive transition is designated as the beginning and end of each cycle. This helps in cases of signal disturbance, as the disturbance is typically a magnitude disturbance and not an additive one. Due to the inductive nature of transformers, which affects the current and causes its sinusoidal activity to become negatively impacted to the point where cyclic analysis is impossible, cyclic histograms are not generated for the current signal. As the voltage is source-driven, it is less susceptible to drift.

The most recent CFG file is loaded and the OSG metadata is extracted. The OSG metadata provides the number of channels, sampling rate, and timestamp, as per the IEEE COMmon format for TRAnsient Data Exchange (COMTRADE) Standard 2013 [18].

#### 2.2.1. Time Synchronization

Due to the physical properties of the transmitted voltage, the signal is never exactly 60 Hz, and the sampling Data Acquisition (DAQ) device will never sample the signal at the exact point of x(t)=0. At the transformer, the frequency can drift by as much as ±0.03 Hz, meaning that the exact time between cycles is inconsistent. Due to this inconsistency, the position of x(t)=0 must be estimated to synchronize each cycle before generating the cyclic histogram. If this frequency drift is not considered, generating the cyclic histogram for one hour of continuous oscillography data is impossible. Each cycle is detected by finding two consecutive negative-to-positive transitions in the sampled waveform x[n]. A window is collected starting with the sample before the first transition and the sample directly after the second transition, and is then processed for time synchronization. An ideal time vector $t_{I}$ is created as a reference, where $t\in [0,1/F_{n}]$ in steps of Δt. A relative time vector $t_{r}$ is generated based on the slope estimated from the windowed signal. The first slope is

(6) $$m_{1}=\frac{x[2]-x[1]}{\Delta t},\;\;\mathrm{and}\;b_{1}=x\left[2\right]-m_{1}t\left[2\right],$$

where $m_{1}$ is the slope between the first two sampled points and $b_{1}$ is the estimated position of the first zero-crossing. The first entry of the relative time vector is

(7) $$t_{r}\left[1\right]=t_{I}\left[1\right]+\frac{b_{1}}{m_{1}}.$$

The end of the windowed signal is used to find the second slope characteristics,

(8) $$m_{1}=\frac{x\left[N_{c}+1\right]-x\left[N_{c}\right]}{\Delta t},b_{2}=x\left[N_{c}\right]-m_{2}\times t_{I}\left[N_{c}\right].$$

The last entry of the relative time vector is

(9) $$t_{r}\left[N_{c}+1\right]=t_{I}\left[N_{c}\right]-\left(\frac{-b_{2}}{m_{2}}-\frac{1}{F_{n}}\right).$$

The rest of the relative time vector is

(10) $$\Delta t_{r}=\frac{t_{r}\left[N_{c}+1\right]-t_{r}\left[1\right]}{N_{c}+1}.$$

With the relative time vector calculated, the values of x(t)=0 now line up with $t_{r}=\left[0,1/F_{n}\right]$. Linear interpolation is used to generate a representation of the sampled waveform x[n] from the relative time vector $t_{r}$ and synchronize it with the ideal time vector $t_{I}$. When a cycle has been collected and synchronized, it is stored to generate the cyclic and residual histograms.

#### 2.2.2. Histogram Generation

**Figure 3:**
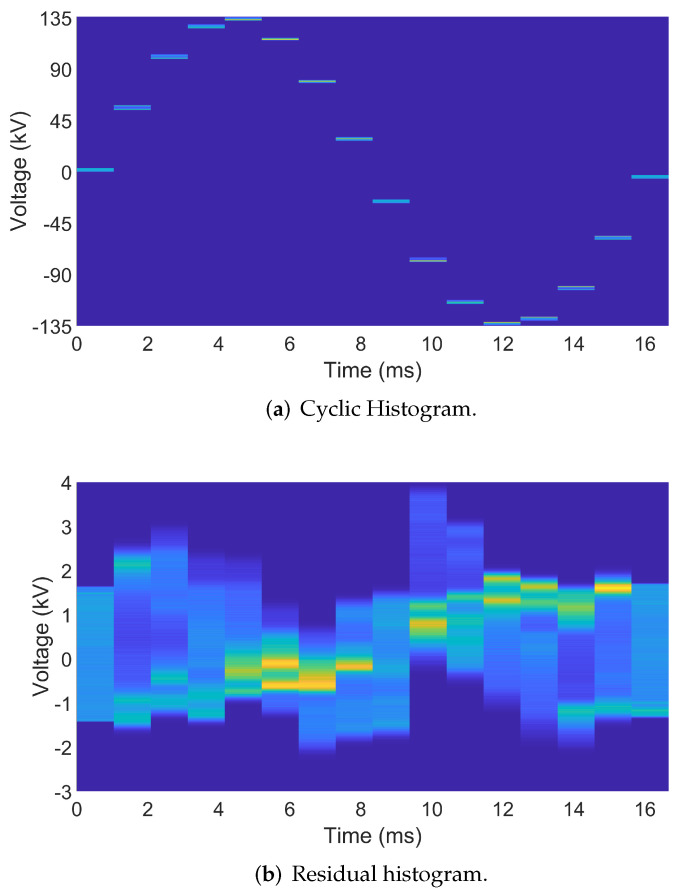
Histograms for one hour of operational data from a 161 kV transformer DFR. The residual histogram’s magnitude range is 7 kV, while that of the cyclic histogram is 270 kV. Note: The hotter or warmer (a.k.a., more red) the color the more often a value occurs across cycles.

A cyclic histogram is a combination of per-sample histograms concatenated to show the quality of the signal over time. For the case of $N_{c}=16$, sixteen histograms are generated for each sample in the nominal cycle and stored in a matrix that represents the cyclic histogram. The global minimum and maximum of all synchronized cycles are used as the bin limits of all histograms to maintain a consistent scale for the cyclic histogram. Each histogram is generated using the *n*th sample of each synchronized cycle. By default, there are 1024 bins per histogram; utility personnel can increase or decrease this resolution as needed. A larger number of bins increases the size of the generated output file. The cyclic histogram is generally unexciting, as seen in Figure 3a. A residual histogram is generated by subtracting the first cycle from the remaining cycles in the record. Subtracting the first cycle accentuates any abnormal behavior(s) present within the processed signal at a per-cycle resolution. The residual histogram corresponding to the cyclic histogram in Figure 3a is presented in Figure 3b. The voltage in Figure 3a is within the range of approximately ±135 kV, while the voltage range in the residual histogram shown in Figure 3b is ±4 kV. This represents an almost 40-fold increase in activity resolution at no additional data cost.

#### 2.2.3. Frequency Histograms

The dominant frequency is calculated using the FFT. The FFT is calculated over all frequencies within $\pm F_{s}/2$. Because the power grid’s frequency is very stable, with an expected maximum deviation of ±0.03 Hz for the 60 Hz fundamental frequency, calculating the FFT over this entire range of frequencies is inefficient. Based on a sampling frequency of 960 Hz, a high-resolution frequency representation (i.e., with a small step size between consecutive frequency values) requires a significant number of zeros (e.g., 1.2 million) to be appended to the end of the time signal. Because the power grid’s frequency is so stable, most of the actionable information is contained within a minimal range of frequencies; thus, most of the resulting frequency response can be “thrown out” without loss of information. Applying zero padding to the time signal wastes computational resources and time, especially when most of the resulting uninformative portions of the frequency response are removed. This problem is addressed by generating a support vector of frequencies centered at 60 Hz and with a Process Bandwidth (PBW) of 0.2 Hz. The PBW can be changed based on the specifics of the DFR or equivalent device and on the preferences or standards of utility personnel. The Discrete Fourier Transform (DFT) of sixty cycles is calculated for only the frequencies specified in the support vector and a step size of thirty cycles between consecutive calculations. This results in the dominant frequency being calculated on a per-second basis, with an overlap of half a second. The FFT is calculated by

(11) $$X\left[f\right]=\sum_{n=1}^{N_{x}}x\left[n\right]\exp\left[-j2\pi ft[n]\right],$$

where

$$f\in \left[F_{n}\pm \frac{PBW}{2}\right]$$

and $N_{x}$ is the total number of samples in the waveform over which the DFT is calculated [19]. The dominant frequency is selected by

(12) $$F_{d}\left(t\right)=\underset{f}{arg max}\left|X\left[f\right]\right|.$$

The output of the dominant frequency is calculated for a sliding 60-cycle window of the recorded waveform, and is then stored and used to generate the frequency histogram. The support of the histogram is the same vector as the PBW calculated in the DFT. The number of cycles per evaluation can be adjusted in the head of the code.

The particular programming platform used allows for Just-In-Time (JIT) run-time compilation directly into machine code via the “Numba” library. Currently, JIT does not support the FFT algorithm; however, it does support the calculation of the described custom DFT. This result is faster and requires far fewer computational resources and time than the zero-padded FFT. Table 1 shows that when JIT is implemented on an i7 workstation with a 10,700 k processor and 64 Gb of Random Access Memory (RAM), the cyclic histogram generation algorithm runtime drops from 603 ms to 13 ms, a speedup of 97.84%. While generation of the residual histogram does not see a speedup, the generation of the frequency and energy histograms is sped up by 88.79%, from 14,317 ms to 1605 ms. This results in an overall runtime of 1630 ms to generate all four histograms for a single line when using JIT acceleration. In our tests, memory utilization never exceeded 300 MB of RAM when reading one hour of data from a single line recording.

### 2.3. Event Types

This section describes the methods employed from the previous section to identify each of the fourteen electrical disturbances, along with the relevant and specific criteria indicating an identification.

#### 2.3.1. Current Transformer Saturation

**Figure 4:**
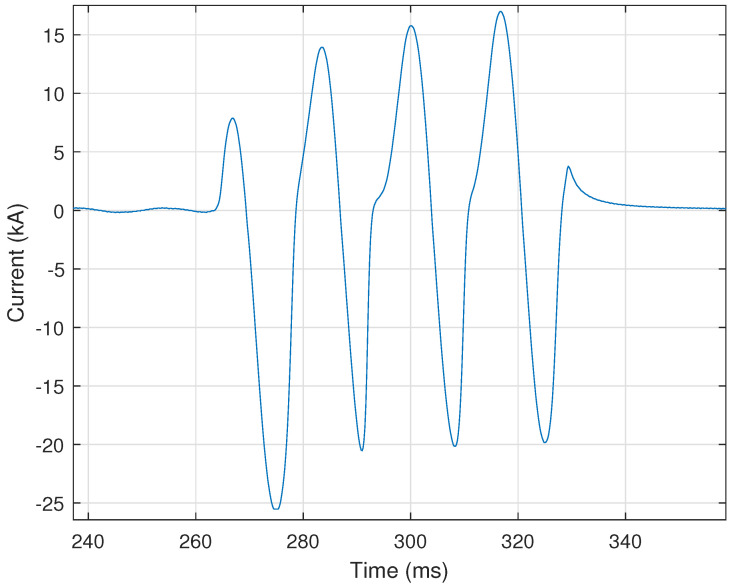
A representative illustration of “kneeing” within a current signal during a CT saturation event.

The first PQ event analyzed is Current Transformer (CT) saturation. A CT is commonly used in relaying or metering applications in high-voltage circuits by producing an alternating current in its secondary winding proportional to the current it is measuring on the high-voltage system. These low-voltage low-magnitude currents are then used as input signals to various instrumentation [20]. CT saturation occurs when the primary current is so high that its core cannot handle more flux. This results in inaccurate replication of the current signal on the secondary winding, which can cause protection relays to operate improperly. A key indicator of CT saturation is a change of slope as the current crosses zero each half-cycle. This change in slope is commonly referred to as “kneeing”. Figure 4 shows a representative illustration of “kneeing”—between 280 ms and 320 ms—within a CT’s current signal.

This work uses the following criteria to determine the occurrence of CT saturation. These criteria are: (i) current exceeding **fifteen** times the continuous current rating of the CT; (ii) presence of DC offset; (iii) the DC offset returning to normal (i.e., 0 Hz) during the fault; (iii) inconsistent spacing between zero crossings; (iv) high third derivative of the current; (v) a high second harmonic current; and (vi) a high third harmonic within the current. These criteria determine the likelihood of CT saturation, as described at the end of this section.

The first step is to determine the presence or absence of a fault. If a fault is detected, processing continues; otherwise it moves to the next event. For the purposes of this work, a fault means that an abnormal current flow has occurred, causing the protective relay(s) to operate and trip the breaker(s). The presence of a fault is determined using the CT ratio defined in the COMTRADE configuration file. The CT ratio is

(13) $$R_{\mathrm{CT}}=\frac{I_{P}}{I_{S}},$$

where $R_{\mathrm{CT}}$ is the turns ratio of the CT, $I_{P}$ is the rated continuous primary current, and $I_{S}$ is the rated continuous secondary current. The CTs used in this work have a continuous rated current of 5 Amperes (A) on the secondary side of the CT; for example, if the CT ratio is $R_{\mathrm{CT}}=240$, then the rated continuous current would be 1200 A on the primary side and 5 A on the secondary side.

If the current exceeds **fifteen** times the continuous current rating of the CT, then a detected fault is high enough to be CT saturation. This threshold was selected based on the recommendations of PQ engineers so as to ensure the selection of only abnormally high faults, as extremely high currents generally indicate CT saturation. Faults not meeting this threshold have a lower chance of being CT saturation. The threshold is provided by

(14) $$\frac{I(n)}{I_{P}}>\tau_{\mathrm{CT}},$$

where *I* is the instantaneous current being analyzed, $I_{P}$ is the rating of the CT on the primary side, $\tau_{\mathrm{CT}}=15$ is the CT saturation threshold, and n=1,2,…,N. The CT saturation threshold was set based on inputs from power utility personnel, and can be changed based on local criteria.

The presence of DC offset is an indicator of CT saturation [20]. DC offset is determined by first calculating the peak value of each cycle’s faulted waveform section. The peaks of the positive and negative half-cycles are then averaged together to provide a value for the offset above or below 0 A. If the maximum of this value exceeds a threshold compared to the nominal peak current, then DC offset is detected in the fault, as provided by

(15) $$\frac{|I_{\mathrm{DC}}|}{{\hat{I}}_{q}}>\tau_{\mathrm{DC}},$$

where $I_{\mathrm{DC}}$ is the maximum DC offset detected during the fault, ${\hat{I}}_{q}$ is the nominal peak current extracted from the first cycle, and $\tau_{\mathrm{DC}}=3$ is the empirically selected threshold for the ratio of DC offset magnitude to the nominal peak current. A loss of DC offset is detected if the offset magnitude is lower at the end of the fault than at the beginning.

The number of samples between zero crossings is then compared to half the nominal number of samples in each cycle calculated using (2), as described in Section 2.1.1. The zero crossing points are calculated as the indices at which the waveform changes sign (i.e., from negative to positive or vice versa). The number of samples between each zero crossing is calculated for every cycle by subtracting the indices accordingly. This number of samples is compared to the nominal value, and is provided by

(16) $$\max\left|N_{Z}\left(k\right)-\frac{N_{c}}{2}\right|>\tau_{Z},\;k=\left(1,2,3,\cdots ,N_{F}\right),$$

where $N_{Z}$ is the number of samples between zero crossings, $N_{c}$ is the nominal number of samples in each cycle, *k* is the index of each cycle, $N_{F}$ is the total number of cycles in the faulted portion of the waveform, and $\tau_{Z}=10$ is the empirically selected threshold for the difference from nominal in the number of zero crossings.

The “kneeing” present in the waveform is detected using a third derivative test. The maximum third derivative present in the first cycle of the waveform (i.e., before the fault) is used as the nominal value. The maximum third derivative of the faulted portion of the waveform is compared to the nominal value, and will be “flagged” if it exceeds a certain threshold, which is provided by

(17) $$\frac{\max|I_{f}^{\prime \prime \prime }\left(n\right)|}{\max|I_{c}^{\prime \prime \prime }\left(n\right)|}>\tau_{D3},$$

where $I_{f}^{\prime \prime \prime }\left(n\right)$ is the third derivative of the faulted current waveform, $I_{c}^{\prime \prime \prime }\left(n\right)$ is the third derivative of the first cycle of the current signal, and $\tau_{D3}=5$ is the empirically selected threshold for the ratio of the fault third derivative to the nominal one.

Finally, the harmonic ratios of the entire current waveform are calculated using Equation (5), as described in Section 2.1.4. An excellent indicator of CT saturation is when the second and third harmonic currents respectively exceed the thresholds of **15%** and **5%** of the fundamental.

All these criteria are combined to provide a confidence level for CT saturation, as follows:

- *High Confidence:* The thresholds are exceeded for the current rating of the CT and the second harmonic current. The thresholds must additionally be exceeded for *three* of the following: DC offset, loss of DC offset, inconsistent spacing between zero crossings, third derivative, or third harmonic current.
- *Medium Confidence:* The threshold is exceeded for the current rating of the CT, but the second harmonic threshold is not exceeded. The thresholds must then be exceeded for *three* of the following: DC offset, loss of DC offset, inconsistent spacing between zero crossings, third derivative, or third harmonic current.
- *Low Confidence:* The threshold is exceeded for the current rating of the CT, but the second harmonic threshold is not exceeded. The thresholds must then be exceeded for *two* of the following: DC offset, loss of DC offset, inconsistent spacing between zero crossings, third derivative, or third harmonic current.
- *Low Confidence (Alternative):* The threshold is not exceeded for the current rating of the CT, but is exceeded for the second and third harmonics. The thresholds must then be exceeded for *two* of the following: DC offset, loss of DC offset, inconsistent spacing between zero crossings, or third derivative.

#### 2.3.2. Analog-to-Digital Converter Clipping

**Figure 5:**
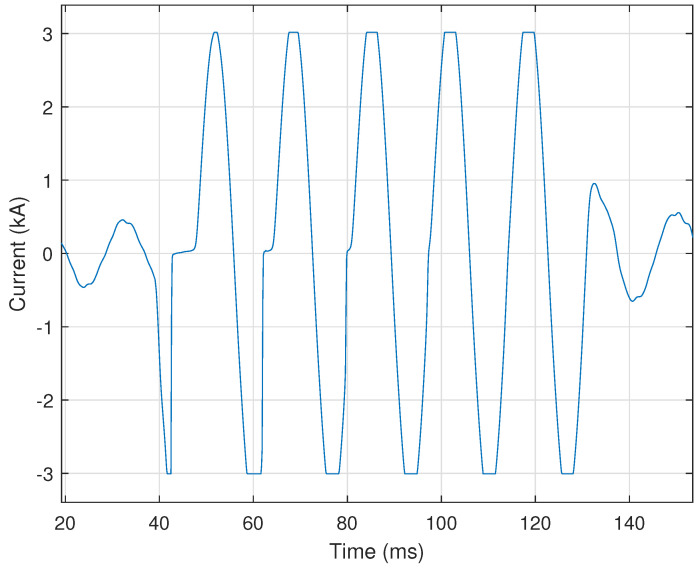
A representative current signal showing Analog-to-Digital Converter (A/D) clipping.

An analog-to-digital (A/D) converter is a device that converts continuously varying analog signals into a binary or digitized sequence. Many electronic devices in substations (e.g., relays and DFRs) utilize A/D converters to record voltage and current signals in a binary format. The power supply rail voltage restricts the range of the digitized scale. If the analog value results in a digitized sequence that exceeds the rail voltage, it will appear “clipped” or “flat-topped” at minimum and maximum values. For substation devices, clipping often appears in current signals during fault events. This results in inaccurate replication of the current signals, which can result in relaying mis-operation. Figure 5 shows the visible clipping at the minimum and maximum values of a current signal’s digitized sequence.

The repetition of equal-magnitude samples within the digitized sequence indicates clipping. First, the index of the absolute maximum of the signal is calculated. The section of the waveform **ten** samples before and **ten** samples after the maximum is then extracted for analysis. If the first derivative of this signal section is equal to zero for more than **four** consecutive samples, then A/D converter clipping is present within the signal.

#### 2.3.3. Induced Transient Noise Due to Switching

**Figure 6:**
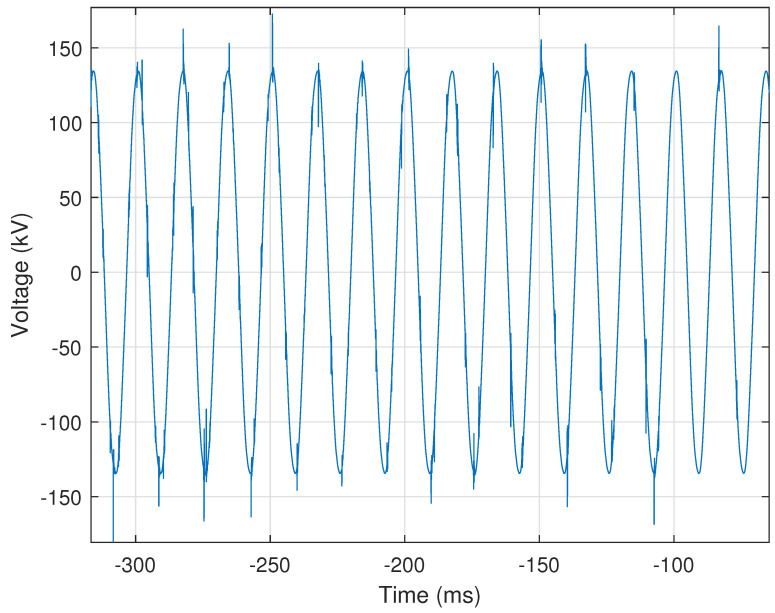
A representative voltage signal showing transient noise due to switching.

When high-voltage devices such as air-break switches are opened to de-energize a bus section, the resulting arcing can induce high-frequency noise upon the voltage or current signals of the electronic monitoring equipment (e.g., PQ monitor). Identifying this induced transient noise can help to determine where signal chokes may need to be installed or where shielding and ground bonding integrity may need to be checked. Figure 6 provides a representative illustration of this transient noise within a voltage signal.

This event is characterized by small random spikes (i.e., noise) throughout the voltage or current signals. Switching-induced transient noise is identified by: (i) overall difference from an ideal waveform; (ii) harmonic content below **5%** of the fundamental; (iii) sudden spikes determined by the first derivative exceeding **10%** of the nominal peak value; (iv) persistence over **five** cycles or more; (v) occurring an average of **once** per cycle; (vi) instances totaling **twenty** or more; and (vii) presence causing individual sample values to exceed the nominal peak signal value occurring ateast **five** times.

The first criterion is determined using the approach described in Section 2.1.5, in which a voltage signal is compared to a reference signal made up of replications of the first cycle. The condition in which the difference between the actual voltage and the reference voltage exceeds a threshold is provided by

(18) $$\frac{{\bar{V}}_{\Delta }}{N}>\tau_{N},$$

where ${\bar{V}}_{\Delta }$ is the mean value of the difference in voltage between the actual and ideal signals, *N* is the total number of samples in the waveform, and $\tau_{N}=30$ is the empirically chosen threshold for this ratio. If the first six criteria are met, induced transient switching is classified with *medium* confidence. If all seven criteria are met, this event type is classified with *high* confidence.

#### 2.3.4. High-Speed Reclosing with Tapped Motor Loads

**Figure 7:**
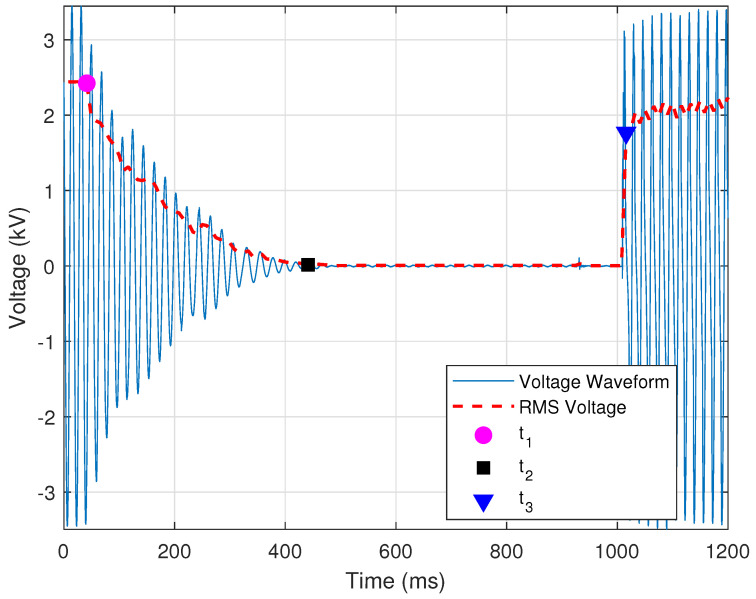
A representation of the case in which the voltage signal *does* decay sufficiently before a successful reclosing operation in the presence of a tapped motor load.

A common practice is to employ high-speed instantaneous reclosing on faulted transmission lines. A large or significant motor load may sometimes be served from stations tapped on the line. In this work, a motor load is considered significant if it is directly served from a high-voltage transmission line (e.g., 161 kV). In such cases, the motors may support the line voltage as they spin down, meaning that residual voltage remains on the line at the time of a high-speed breaker reclosing operation. In larger machines, the residual voltage may require up to five seconds to decay [21]. Because this residual voltage is unlikely to be in phase with the system voltage, the result can be a failed reclosing attempt by the line breakers and damage to the motors. Thus, it is important to identify lines where high-speed reclosing must be delayed to allow the voltage to sufficiently decay before reclosing. Figure 7 shows a voltage signal in which sufficient time has passed to enable the voltage signal to decay to a point after which the reclosing operation can be completed.

Such an event is identified by determining whether the operation is high-speed reclosing. For the purposes of this work, a reclosing operation is designated as high-speed if it is “blind” (i.e., without supervision or checks) and occurs within thirty cycles of the initial current interruption by the breaker [21]. Identification of reclosing with tapped motor loads is achieved by determining the sample points at which: (i) the voltage signal begins to decay; (ii) the voltage signal reaches zero; and (iii) the reclosing operation occurs. The time between these three points determines whether the reclosing operation is a high-speed one. In this work, and as shown in Figure 7, these three sample points are designated as $t_{1}$ (magenta circle), $t_{2}$ (black square), and $t_{3}$ (blue triangle), respectively. The location of these three sample points is determined using the RMS signal, which is calculated using Equation (3), as described in Section 2.1.2, and is shown in Figure 7 as a broken red line. For this event, the RMS window is set to half the number of samples in each cycle (i.e., $N_{c}/2$).

The point $t_{1}$ is the time at which the RMS voltage first decays below a threshold, and is determined by

(19) $$\frac{\bar{V}\left(t\right)}{{\bar{V}}_{q}\left(t\right)}<\tau_{S},$$

where $\bar{V}$ is the RMS of the voltage, ${\bar{V}}_{q}$ is the nominal RMS voltage as determined from the first cycle, and $\tau_{S}=0.9$ is the empirically selected threshold for the sag in voltage indicating the start of a decay. The point $t_{2}$ is determined as the time a which the voltage decays enough to be considered approximately zero. An empirical threshold of $\tau_{0}=0.01$ was used as the threshold below which the RMS voltage must fall to be considered zero. If this condition is not met, then $t_{2}$ is when the RMS voltage is at its minimum. The RMS voltage must decay to below **50%** of the nominal value in order for the process to continue.

The voltage decay portion is the RMS voltage between times $t_{1}$ and $t_{2}$, and is designated here as ${\bar{V}}_{D}$. The median (i.e., middle value) of ${\bar{V}}_{D}$ must be lower in magnitude than the voltage at time $t_{1}$ and higher than the voltage at time $t_{2}$. In addition, the mean of the first derivative of ${\bar{V}}_{D}$ must be negative to indicate a downward slope or a decrease in voltage. The maximum first derivative of the voltage decay must be less than a threshold to ensure that the voltage decay is not sudden. This condition is provided by

(20) $$\frac{\max|{\bar{V}}_{D}^{\prime }|}{{\bar{V}}_{q}}<\tau_{l},$$

where ${\bar{V}}_{D}^{\prime }$ is the first derivative of the decaying portion of the RMS voltage, ${\bar{V}}_{q}$ is the nominal RMS voltage, and $\tau_{l}=0.5$ is the empirically selected threshold for the maximum first derivative of the decaying voltage. The point $t_{3}$ is when the RMS voltage increases by **30%** of nominal value in one RMS sample. This condition is determined by the first derivative of the RMS signal, as provided by

(21) $$\frac{\max|{\bar{V}}_{S}^{\prime }|}{{\bar{V}}_{q}}>\tau_{U},$$

where ${\bar{V}}_{S}^{\prime }$ is the first derivative of the portion of the RMS voltage after time $t_{2}$, ${\bar{V}}_{q}$ is the nominal RMS voltage, and $\tau_{S}=0.3$ is the empirically selected threshold for the minimum first derivative of the reclosing voltage. Time $t_{3}$ is when reclosing occurs and the voltage is restored.

**Figure 8:**
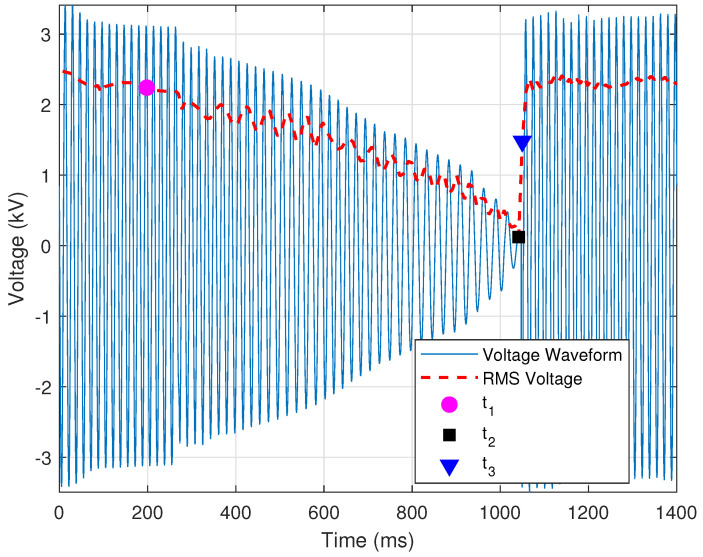
A representation of a case in which the voltage signal *does not* decay sufficiently before a successful reclosing operation in the presence of a tapped motor load.

Thus far, the provided criteria aim to classify the event as normal reclosing with a tapped motor load. Figure 7 is a normal event with sufficient time between $t_{2}$ and $t_{3}$. If the time between these two points is insufficient, the event is “flagged” as needing attention. The condition that defines a high-speed reclosing operation is provided by

(22) $$t_{3}-t_{2}>\tau_{\mathrm{HS}},$$

where $t_{2}$ is the time at which the voltage first decays to zero, $t_{3}$ is the time at which the voltage is restored, and $\tau_{\mathrm{HS}}=30\;\mathrm{cycles}$ is the threshold for the minimum recommended time for which the voltage must be zero before reclosing [21]. Figure 8 shows a case in which the minimum time for which the voltages need to be zero has not been satisfied.

#### 2.3.5. DC Offset

DC offsets in analog channels are a common issue; when they are large enough, they can negatively impact RMS calculations. A large DC offset is accounted for by recalibration of the corresponding monitoring or recording device. Automated calculation of DC offset allows utility personnel to prioritize recalibration of those devices associated with the largest amounts of DC offset. The DC offset event is characterized by an asymmetry between the positive and negative half-cycles of a voltage or current signal.

**Figure 9:**
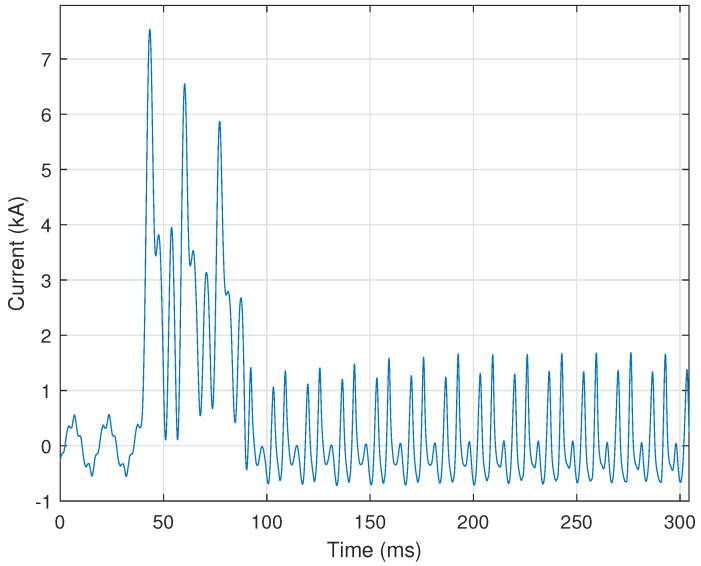
Representative illustration of a large DC offset—from 40 ms to 90 ms—within a current signal.

**Figure 10:**
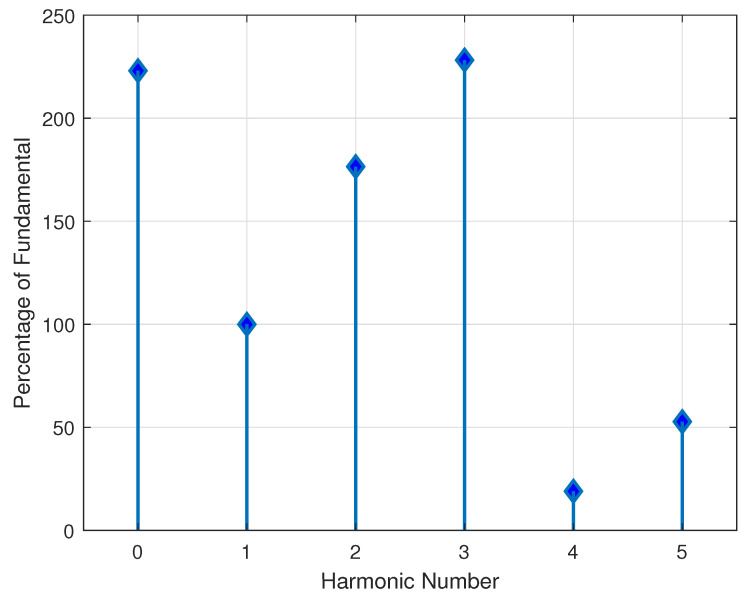
Illustration of the zeroth through fifth harmonic ratios of the current signal shown in Figure 9.

The presence and amount of DC offset are determined using time and frequency domain analysis. In the frequency domain, a DC offset is present if the magnitude of the 0 Hz frequency component is greater than 50% of the magnitude at the fundamental frequency component (i.e., 60 Hz in the United States). Mathematically, this condition can be expressed as

(23) $$\frac{X_{0}}{X_{1}}>\tau_{f},$$

where $X_{0}$ is the magnitude of the 0 Hz frequency component, $X_{1}$ is the magnitude at the fundamental frequency component, and $\tau_{f}=0.5$ is empirically selected as the minimum ratio for the fundamental frequency. Figure 9 provides a representative illustration of a current signal in which a large amount of DC offset is present from 40 ms to 90 ms. Figure 10 shows the magnitude of the zeroth through fifth harmonic of the current signal shown in Figure 9. In this case, the 0 Hz frequency component is over two times larger than the fundamental frequency component (i.e., the first harmonic), and would be “flagged” as a DC offset event. Interestingly, the third harmonic indicates that another disturbance is present within the recorded signal of Figure 9.

If frequency domain analysis identifies a DC offset event, then time domain analysis is performed as a validation step. Time domain analysis is conducted by computing the mean over each cycle within the recorded signal. If the mean value of a given cycle is zero, then no DC offset is present within that cycle. This is because the areas under the positive and negative portions of the cycle would negate each other. However, if the mean of the selected cycle exceeds 50% of the nominal signal’s peak value, the DC offset event “flag” is set again. The amount of DC offset returned by the automated process is

(24) $$\underset{i}{arg max}\mu_{i},$$

where $\mu_{i}$ is the mean value of the *i*th cycle within the signal being processed.

#### 2.3.6. Motor Starting

**Figure 11:**
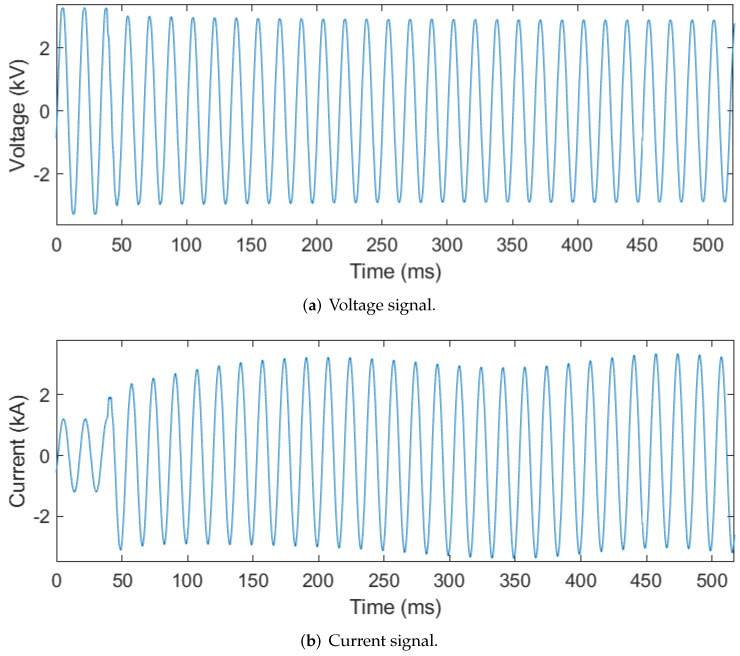
Voltage and current signals showing signal characteristics associated with a motor starting event.

Instantaneous increases in current may be due to faults, motor starts, transformer energizations, or other events. Signatures within the recorded signals can be used to distinguish and classify each event. PQ disturbances can then be correlated by event classification. In the case of motor starting, the voltage sags, and the current can increase to five to six times its rated value [22]. It is challenging to set protective relays in such a way as to enable recognition of a motor starting event rather than recognizing the event as a fault in the system. The automated process described in this section is developed assuming that the corresponding relays are properly set to ensure that they do not trip open when motor inrush current is present. Figure 11a,b shows representative illustrations of motor starting voltage and current signals, respectively.

The automated process checks for a voltage sag below 95% of the signal’s nominal RMS value and a current spike to twice the CT’s rated value (as determined by (13)). If both of these conditions persist for at least ten consecutive cycles, then the first indicator of motor starting is identified. The persistence of both conditions for ten or more consecutive cycles distinguishes motor starting events from a fault condition, which typically occurs for only several cycles before the relay trips open the breaker. Motor starting events are additionally associated with a frequency response that is low in harmonic content. Thus, if none of the voltage or current signals’ harmonics exceed **15%** of the magnitude of the fundamental frequency components, then the second indicator of motor starting is identified. As motors are three-phase devices, the final indicator for motor starting is that all three conditions (i.e., voltage sag, current spike, and harmonics below 15% of the fundamental) occur in all three phases.

#### 2.3.7. Variable-Frequency Drive Motor Starting

**Figure 12:**
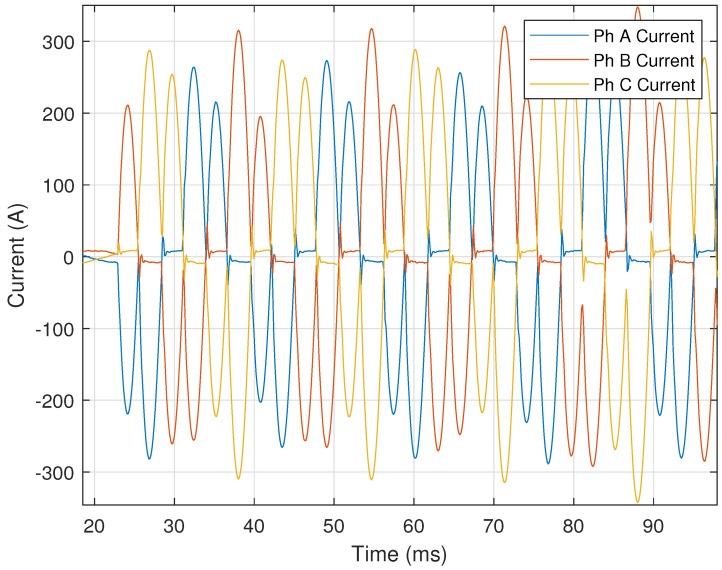
A illustration showing the distinct current signal generated during a six-pulse VFD motor starting event.

Certain motors utilize electronic starting (e.g., Variable Frequency Drives, or VFDs) to bring the motor up to speed in a controlled manner in order to limit voltage supply disturbance(s). VFDs produce unique harmonic patterns, which allow these events to be easily identified by our automated process. When a VFD motor starts, it creates a very distinct current signal. A representative illustration of this distinct current signal can be seen in Figure 12.

**Figure 13:**
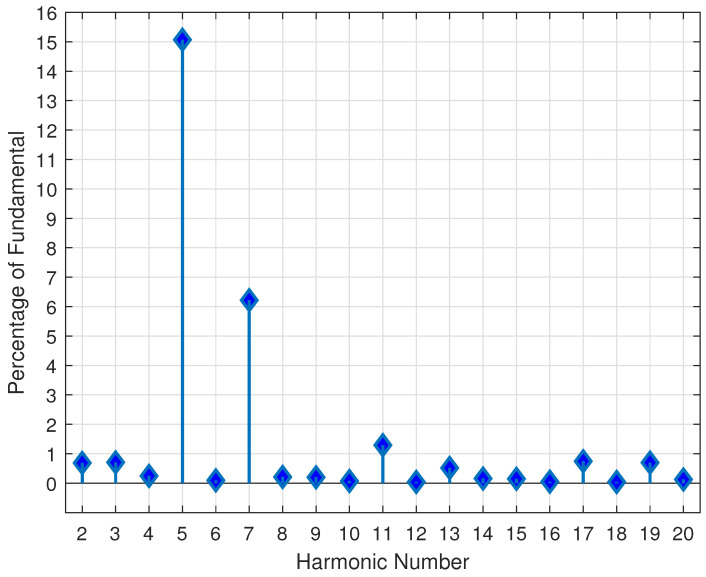
The harmonic ratios calculated from the current signal of the six-pulse VFD motor start event shown in Figure 12.

In Figure 12, each phase has two pulses per half-cycle. The number of pulses per half-cycle indicates the type of VFD, e.g., six-pulse, twelve-pulse, etc. VFD motor starting events are identified by counting the number of times the current signal drops below **50%** of each cycle’s maximum value. Two pulses in each half cycle of a current signal for each phase (e.g., Figure 12) would indicate a six-pulse VFD. The number of pulses for the drive is provided by

(25) $$N_{p}=\frac{3}{2}\times mode\left(K\right),\;K>2,$$

where *K* is the number of times the current crosses 50% of each cycle’s maximum value every half-cycle and mode(K) refers to the most frequently occurring value of *K*. The current must cross the threshold more than **two** times for at least **eight** cycles during the event in order to be considered VFD motor starting. Harmonic analysis is conducted after $N_{p}$ is calculated, as VFD motor starting events result in dominant harmonics on either side of an integer multiple of $N_{p}$. Figure 13 shows the harmonics for the six-pulse (i.e., $N_{p}=6$) VFD motor starting event illustrated in Figure 12. The fifth and seventh harmonics are the two most dominant harmonics, and occur on either side of the sixth harmonic (equal to that of $N_{p}=6$). The value of $N_{p}$ is validated by ensuring that the dominant harmonics are at least **five** times larger than the value of the harmonics at integer multiples of the $N_{p}$. This validation check is performed by

(26) $$\frac{H_{kN_{p}\pm 1}}{H_{kN_{p}}}>\tau_{V},\;\left(k=1,2,3,4\right),$$

where $N_{p}$ is the number of pulses in the VFD, $H_{kN_{p}}$ is the harmonic at an integer multiple of $N_{p}$, *k* is an integer, and $\tau_{V}=5$ is the empirically determined threshold for the ratio of the dominant harmonics to those at integer multiples of $N_{p}$. If Equation (26) is satisfied, then the number of predicted pulses is deemed correct. Finally, the event is identified as a VFD motor starting as long as all three currents (i.e., phase A, B, and C) increase over the event’s duration.

#### 2.3.8. Melting Fuse

**Figure 14:**
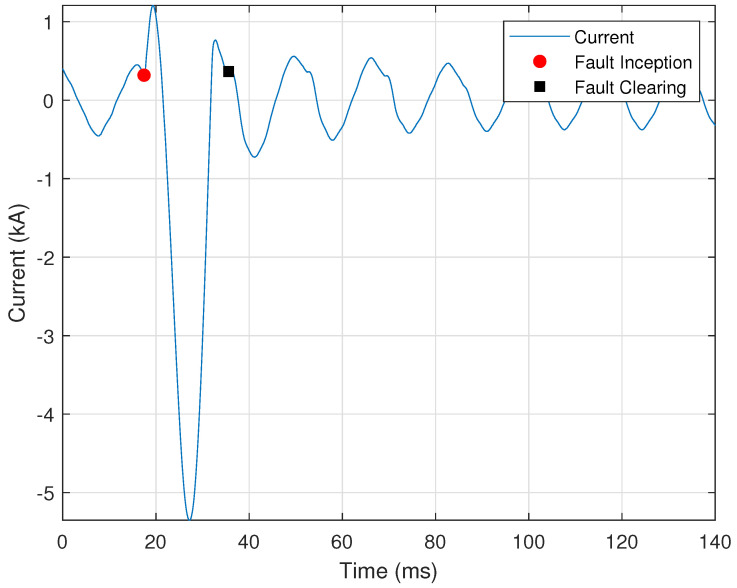
A current signal during a fuse melting event lasting just over one cycle.

Unlike a breaker, a blown (melted) fuse requires utility personnel to replace it physically; thus, it is helpful to distinguish fuse faults from breaker faults. These two faults are characterized by the speed at which the fault is cleared. Breakers require two or more cycles to clear a fault, while fuses require less than two cycles. Figure 14 shows an example of a fuse melting event cleared in just over one cycle.

The key to automated identification of fuse melting events is to accurately determine the fault’s inception and clearing points. A fuse melting event occurs if the total clearing time is less than one and a half cycles, and is determined by

(27) $$|t_{I}-t_{C}|<\tau_{c},$$

where $t_{I}$ is the inception point, $t_{C}$ is the clearing point, and $\tau_{c}=1.5$ is the threshold for the maximum fuse clearing time.

Automated identification of a fuse melting event is initialized by determining whether the event persisted for at least a quarter of a cycle and whether the current reached at least twice its nominal value over the event’s duration. The cycle before and just after the portion associated with these two conditions is then analyzed one half-cycle at a time to determine the fault inception and clearing points. The three possible approaches used to determine these points are: (i) a sign change in the first derivative; (ii) a sudden increase in the second derivative; and (iii) the current signal’s zero crossings.

The first derivative approach is implemented using Equation (4), as described in Section 2.1.3. A sign change in the first derivative before or after the spike in current indicates the fault inception and clearing points. This approach determines the inception and clearing points of the fuse melting event shown in Figure 14, where the red circle indicates the fault inception point and the black square indicates the fault clearing point.

If the first derivative approach is unsuccessful, i.e., a sign change in the first derivative does not exist, then the second derivative is used, as described in Section 2.1.3. The condition for a large second derivative is provided by

(28) $$\frac{\max|I^{\prime \prime }\left(n\right)|}{{\hat{I}}_{q}}>\tau_{D2},$$

where $I^{\prime \prime }\left(n\right)$ is the second derivative of the current signal, ${\bar{x}}_{c}$ is the nominal peak current, and $\tau_{D2}=0.02$ is the empirically selected threshold for the minimum ratio of the second derivative of the current to the nominal value. This approach was used to determine the fault inception point of Figure 1, as described in Section 2.1.3.

Suppose that the second derivative approach is unsuccessful, i.e., the minimum threshold is not met. In this case, the fault inception and clearing points are assumed to be zero crossings just before and after the current spike. After the fault inception and clearing points are determined, Equation (27) is used to determine whether the fault was short enough in duration to be a melted fuse.

#### 2.3.9. Ferroresonance

**Figure 15:**
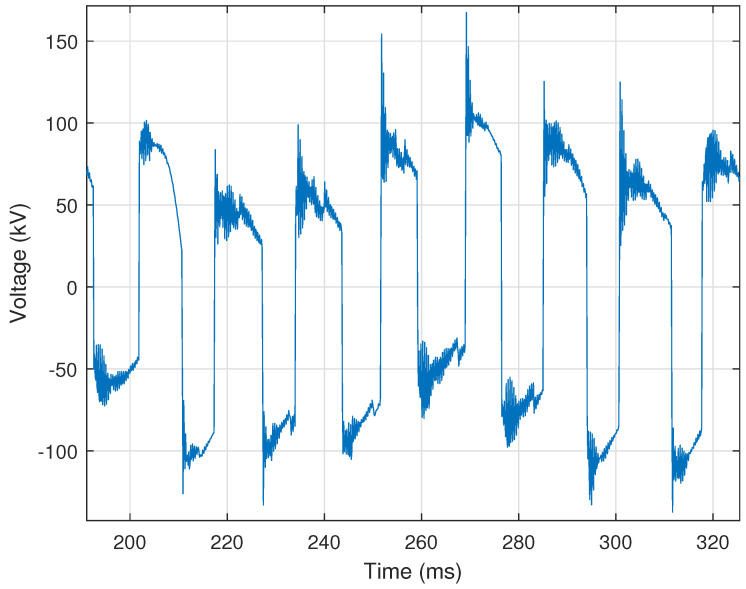
Illustration of a voltage signal collected during a ferroresonance event.

Ferroresonance is electric circuit resonance that occurs when a circuit containing nonlinear inductance is fed from a source that has a series capacitance connected to it. In a transmission system, ferroresonance can occur when a breaker with grading capacitors is used to de-energize a bus with magnetic Voltage Transformers (VTs) connected to it. The described scenario presents a serious safety risk to utility personnel and damage risk to equipment, as severe overvoltages can occur despite the open breaker. Ferroresonance manifests in voltage signals, causing the signals to take on a square wave-like shape/appearance. Figure 15 provides a representative illustration of the square wave appearance that a voltage signal can take on due to ferroresonance. Another characteristic of ferroresonance events is that the current is usually zero during the event, as the line is de-energized; however, depending on the location of the recording device, the current can be recorded as a nominal signal.

Ferrroresonance events are identified using three criteria: (i) a large difference between discrete samples in the voltage signal; (ii) the behavior continuing for a certain number of cycles, and often remaining enough during that time; (iii) significant harmonic content in the voltage signal; and (iv) the current signal being recorded as zero or a nominal waveform.

The first criterion is met if the first derivative of the voltage signal exceeds 50% of nominal peak voltage, as provided by

(29) $$\frac{|V^{\prime }\left(n\right)|}{{\hat{V}}_{q}}>\tau_{F},$$

where $V^{\prime }\left(n\right)$ is the first derivative of the voltage signal, ${\hat{V}}_{q}$ is the nominal peak voltage, and $\tau_{F}=0.5$ is the empirically selected threshold for the minimum ratio of the first derivative of the voltage to the nominal value. The second criterion is met if this threshold is exceeded a minimum of **five** times, occurs at least every **three** cycles, and occurs for a length of at least **five** cycles. The third criterion is met if one of the harmonic currents is greater than **5%** of the fundamental. Finally, the fourth criterion is met if the RMS current is recorded as zero or the current signal is nominal, characterized by a small number of first derivative sign changes. This nominal condition is provided by,

(30) $$\frac{N_{I}}{N}<\tau_{I},$$

where $N_{I}$ is the number of first derivative sign changes in the current as calculated using Equation (4), *N* is the total number of samples in the waveform, and $\tau_{I}=0.3$ is the empirically selected threshold for the ratio of sign changes to total samples.

#### 2.3.10. Capacitor Bank Switching

**Figure 16:**
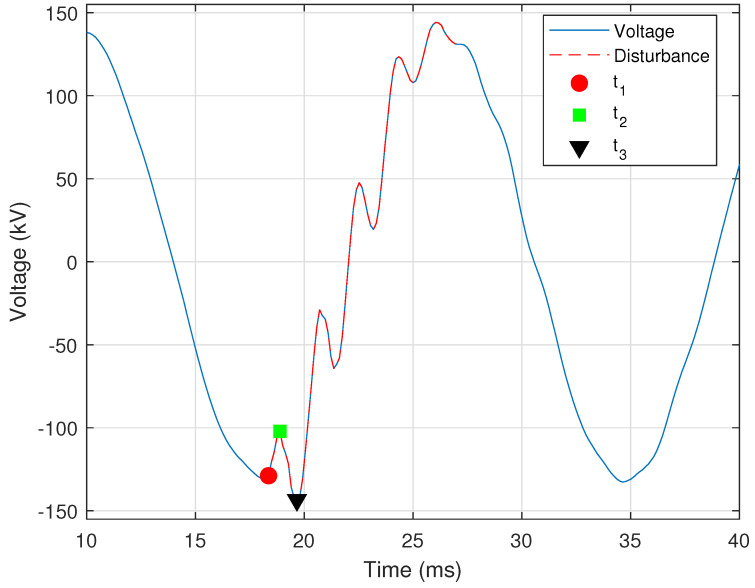
Illustration of a voltage signal collected during a capacitor switching event.

One of the most common events in a power system is capacitor bank switching. Capacitor bank switching induces temporary voltage transients that can create PQ events. A typical capacitor bank switching transient is characterized by a quick depression of the voltage signal towards zero, followed by an overshoot and subsequent transient disturbance lasting approximately one cycle as the system returns to a steady state. These voltage transients may be recorded by devices connected to the same bus as the capacitor bank or by devices connected to a different bus. Based on this fact, the presented automated process is designed to identify capacitor switching for both recording device connection scenarios. Figure 16 shows an example of capacitor bank switching, in which the broken red line highlights the portion of the recorded signal associated with the event.

Capacitor banks are simultaneously switched on in all three phases in a power transmission system. Although Figure 16 shows only a single phase, the other two-phase voltage signals are similar in appearance; however, due to the 120° phase difference between each of the three signals, they will not be identical (i.e., the switching event occurs at different points of the corresponding phase’s sinusoidal signal). The disturbance is located within the signal using the first cycle as a reference, as described in Section 2.1.5 and shown in Figure 2. The condition for the difference between the actual and ideal voltage signals is provided by

(31) $$\frac{|V_{\Delta }|}{{\hat{V}}_{q}}>\tau_{\Delta },$$

where $V_{\Delta }$ is the difference between the actual and ideal voltage signals, ${\hat{V}}_{q}$ is the nominal peak voltage value, and $\tau_{\Delta }=0.02$ is the empirically selected threshold for this ratio. When the presence and location of the disturbance have been determined, the disturbance’s duration is calculated to ensure that it does not exceed **two** cycles. The voltage signal’s peak values must satisfy one of these two criteria: (i) one peak is **2%** above nominal value and no more than one peak is **10%** above the nominal value; and (ii) precisely two peaks **10%** above the nominal value occur in neighboring cycles.

The next step is to determine the three characteristic points highlighted on the waveform of Figure 16, which are designated as $t_{1}$ (red circle), $t_{2}$ (green square), and $t_{3}$ (black triangle). These points are indicative of a capacitor switching event. First, the portion of the voltage signal one half-cycle before and one half-cycle after the highest peak value is extracted, designated as $V_{O}$. The point $t_{1}$ is determined as the first point in which the voltage signal’s first derivative exceeded a certain threshold, as provided by

(32) $$\frac{|V_{O}^{\prime }\left(n\right)|}{{\hat{V}}_{q}}>\tau_{O},$$

where $V_{O}^{\prime }\left(n\right)$ is the first derivative of the overvoltage cycle of the voltage signal, ${\hat{V}}_{q}$ is the nominal peak voltage value, and $\tau_{O}=0.02$ is the empirically selected threshold for this ratio. The first occurrence of this condition is determined to be $t_{1}$. Point $t_{2}$ occurs at the lowest point of the signal, at which the magnitude of voltage signal has dropped below **90%** of the nominal peak value. Point $t_{3}$ is then determined as the time index of the highest peak of the voltage signal $V_{O}$.

The location of these three characteristic points is then validated using the following three checks: (i) the voltage magnitudes at these points are the expected values; (ii) there is a nominal number of samples between the overvoltage and the peak before it; and (iii) the waveform slope is reversed at $t_{1}$. For the first check, the expected voltage magnitudes at $t_{1}$, $t_{2}$, and $t_{3}$ must follow the inequality provided by

(33) $$|V_{t_{2}}\left|<\right|V_{t_{1}}\left|<\right|V_{t_{3}}|,$$

where $|V_{t_{1}}|$, $|V_{t_{1}}|$, and $|V_{t_{3}}|$ are the voltage magnitudes at times $t_{1}$, $t_{2}$, and $t_{3}$, respectively. The second check is that the peak before must be approximately equal to $N_{c}/2$ samples before the overvoltage peak, as determined by

(34) $$\frac{N_{\mathrm{PB}}-N_{c}/2}{N_{c}}<\tau_{P},$$

where $N_{\mathrm{PB}}$ is the number of samples between the overvoltage peak and the peak before it, $N_{c}$ is the number of samples in each cycle, and $\tau_{P}=0.1$ is the empirically selected threshold for this ratio. Finally, the third check is validated using (4), as described in Section 2.1.3. If the first derivative of the voltage signal leading up to $t_{1}$ is of the sign opposite to the voltage’s first derivative between $t_{1}$ and $t_{2}$, then the third check is met. When all these criteria are met for one of the three voltage phases, the other two phases are analyzed to ensure that some form of disturbance is present.

#### 2.3.11. Lightning Strikes

**Figure 17:**
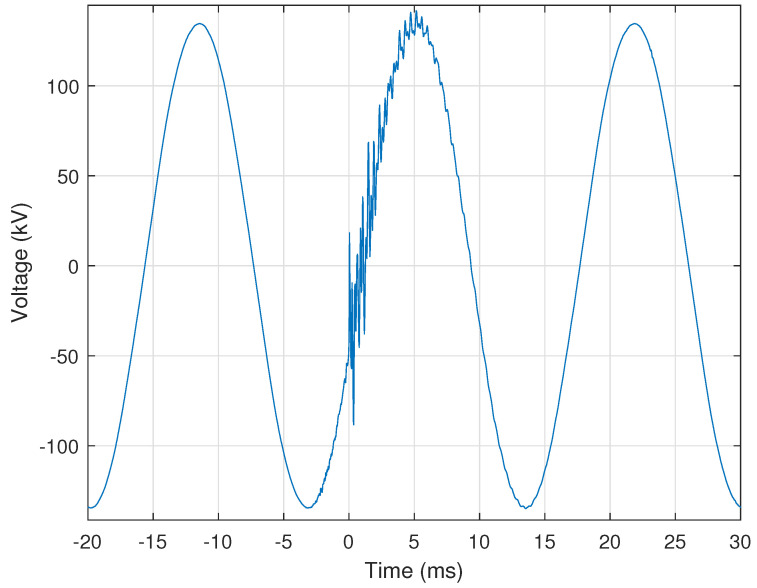
Illustration of a voltage signal collected during a lightning strike event.

Transient overvoltages due to lightning strikes on a transmission line are typically impulses with a rise and decay time in the microseconds. Due to the limitations of instrument transformers to pass these high frequencies and instrumentation sampling rates, lightning strike events are not readily identified. A representative voltage signal that includes a lightning strike event is shown in Figure 17.

First, the automated identification process attempts to identify the event as a capacitor bank switching event (Section 2.3.10), then a melting fuse event (Section 2.3.8). These steps are taken to ensure that a lightning strike event is not incorrectly identified as either of these events, which, although similar to a lightning strike, are easily distinguished from both it and one another. Supposing that the event is not identified as capacitor bank switching or a melting fuse, the disturbance is isolated from the overall signal using the same method provided in Equation (31) for isolating the disturbance caused by a capacitor bank switching event. The disturbance isolation process is repeated for each lightning strike and the longest strike duration is checked to ensure that it does not exceed **one** cycle. If more than **five** disturbances are isolated, then the event is not identified as a lightning strike. In all the processed data, lightning did not strike more than three times during a single recording. Thus, as long as no more than three lightning strike disturbances are isolated, the automated process identifies the event as a lightning strike and returns the number of strikes along with the disturbance’s duration in seconds.

#### 2.3.12. Harmonic Resonance

**Figure 18:**
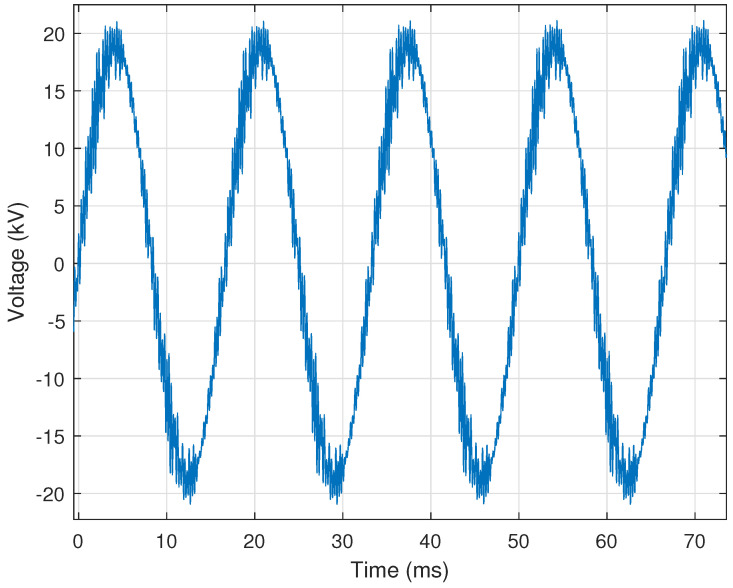
Illustration of a voltage signal collected during a harmonic resonance event.

Power systems have natural frequencies that are a function of the system’s inductive and capacitive impedance. When a nonlinear load on the power system, such as a VFD, generates a frequency that is a natural frequency of the power system, i.e., a multiple of the fundamental frequency, then a resonance condition can result. This resonance can subject equipment to overvoltages or currents, resulting in equipment failure or misoperation. Thus, it is crucial to detect harmonic resonance conditions quickly to ensure that appropriate and necessary actions can be taken to correct the problem(s). Figure 18 shows an example of harmonic resonance on an operationally recorded voltage signal.

Harmonic resonance is characterized by high-frequency content in the voltage signals. Based on this information, the automated identification process first calculates the Total Harmonic Distortion (THD) of the voltage signal using

(35) $$V_{\mathrm{THD}}=\frac{\sqrt{\sum_{i=2}^{M}{\left|H_{i}\right|}^{2}}}{H_{1}},$$

where $H_{i}$ is the *i*th harmonic, $H_{1}$ is the fundamental frequency, M=100 is the total number of harmonics used for the calculation, and |•| denotes the magnitude [23]. If the THD is greater than **8%** of the fundamental frequency, then the process continues; otherwise, it moves on to the next event category. A value of 8% was empirically selected, which can be adjusted as more data become available or based on the specifics of the power system.

If the THD threshold is satisfied, then the automated identification process determines whether or not at least the sixth or one of the higher harmonics is more than **5%** of the fundamental frequency’s magnitude. If this is the case, then the sign changes in the first derivative are calculated for each cycle using (4), as described in Section 2.1.3. The number of first derivative sign changes in each cycle must be at least **10%** of the samples in each cycle $N_{c}$ and must occur across **three** cycles. The automated process identifies the event as harmonic resonance if all criteria are met.

#### 2.3.13. Improper Voltage Transformer Secondary Grounding

Using a single solid grounding point on an instrument VT’s secondary is good design practice [24]. Otherwise, the result may be incorrect secondary voltage signals in both magnitude and angle, leading to the misoperation of protective relays. This can be exacerbated when faults occur on the lines protected by these relays.

**Figure 19:**
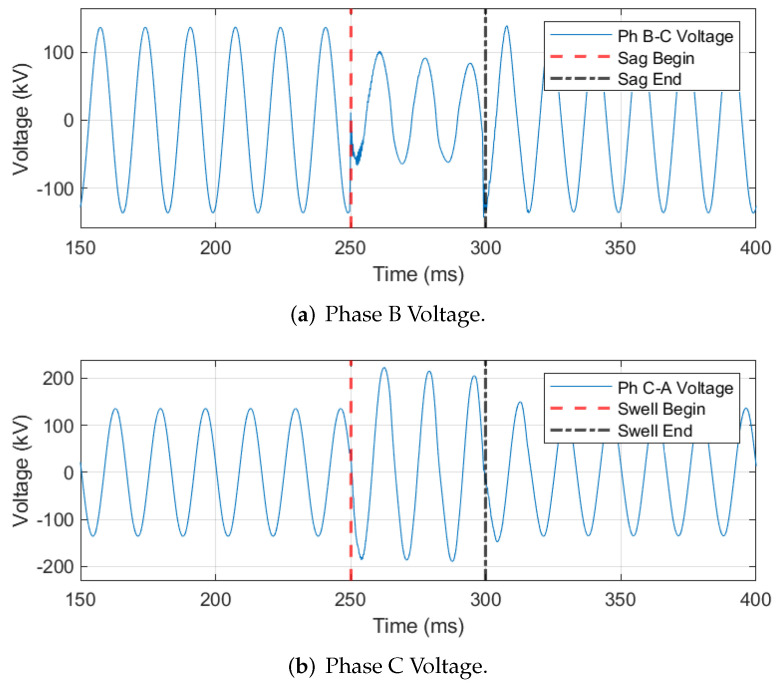
Representative voltage signals indicating improper VT secondary grounding due to the simultaneous presence of a voltage sag and swell in two phases.

An indicator of improper VT secondary grounding is when one voltage phase is sagged while another is swelled. Figure 19 provides a representative example of this indicator in which the Phase B voltage signal is experiencing a sag from 250 ms to 300 ms (Figure 19a). In contrast, the Phase C voltage signal experiences a swell over the same period (Figure 19b). Automated identification of improper VT secondary grounding is facilitated by determining whether a simultaneous voltage sag and swell exists on two of the three voltage phases. In this work, a sag occurs when one of the voltage signal’s peaks *falls* below the nominal peak voltage by more than **5%** and a swell occurs when one of the voltage peaks *rises* above the nominal peak by more than **5%**. The phase angle between the sagged and swelled voltage phases is calculated using

(36) $$\theta =\cos^{-1}\left(\frac{V_{\alpha }\cdot V_{\beta }}{|V_{\alpha }\left|\right|V_{\beta }|}\right),$$

where $V_{\alpha }$ and $V_{\beta }$ are two of the three faulted voltage phasors, · denotes the dot product, and θ is the phase angle between $V_{\alpha }$ and $V_{\beta }$. The phase angle is calculated between phases A to B, B to C, and A to C. In a balanced system, the nominal angle between two voltage phases is 120° [25]. If the phase angle deviates from this 120° nominal angle by more than **5°**, then the event is identified as an improper VT secondary grounding event.

#### 2.3.14. Incipient Capacitive Voltage Transformer Failure

**Figure 20:**
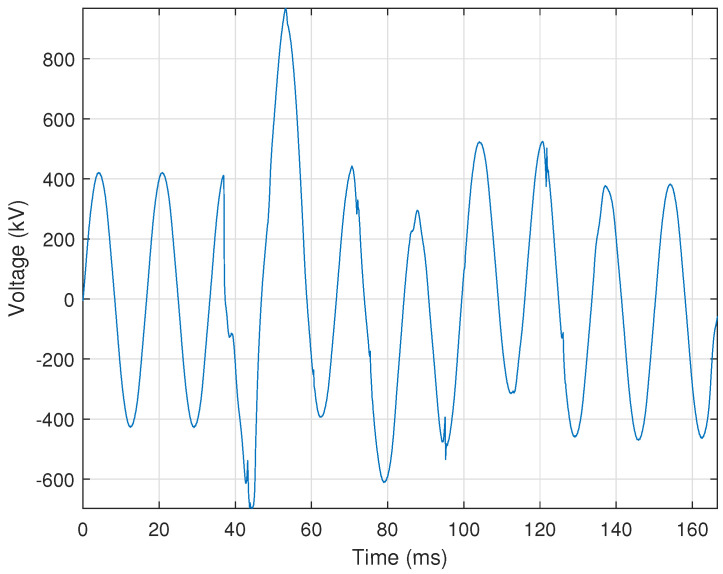
Illustration of a voltage signal showing an incipient CVT failure event.

Capacitive Voltage Transformers (CVTs) supply voltage to protective relays; thus, it is imperative that the CVT measures voltage accurately. If a catastrophic CVT failure results in a complete loss of this voltage, the affected relays detect the loss using Loss of Potential (LOP) logic and act accordingly [26]. However, relays are not equipped to detect a CVT showing early signs of failure by providing incorrect data when it has not yet failed to provide the supply voltage. The automated identification process developed here is designed to detect early indicators of impending CVT failures in order to facilitate proper actions by utility personnel or equipment. Additionally, a CVT failure poses a significant safety risk to any utility personnel who happen to be nearby when it fails. The voltage signal shown in Figure 20 represents the early indicators of an impending (incipient) CVT failure.

The first indicator of an incipient CVT failure event is that one of the voltage signal’s peaks contains a rise or fall of more than **10%** of the nominal peak value persisting for at least **three** cycles. The second incipient CVT failure indicator is that the disturbance portion of the voltage signal differs from its corresponding nominal signal by more than $\tau_{\Delta }=0.02$, as introduced in Section 2.1.5 and implemented in (31). Because CVTs are single-phase devices, incipient CVT failure occurs only in one phase, which is a differentiating factor between other events. Finally, the current signal is analyzed to ensure that no disturbance is present, as this event type is specific to voltage signals.

## 3. Results

The performance of the developed rule-based automated electrical disturbance identification process was assessed using a dataset comprising 160 total event records collected by field devices operating in a power utility’s transmission system. This dataset contains approximately ten records for each of the discussed events. The dataset includes events with undisturbed voltage and current signals as well as single-phase and multi-phase events. Each phase of every single-phase event was processed, tripling the size of the associated event’s dataset. False positive and false negative event identifications were counted as incorrect or misidentifications. If a signal did not contain one of the listed electrical disturbances and the automated process did not identify it as a disturbance, it was counted as a correct result. Overall automated identification results are presented in Table 2 for each of the fourteen event types. Table 2 provides the number of events analyzed, the number of events identified correctly, and the accuracy for each event type as a percentage.

### 3.1. Results: Current Transformer Saturation

The accuracy of the automated process in determining CT saturation was 96.67% (i.e., correctly identifying 464 out of 480 total signals processed). The test for CT saturation proved to be challenging due to the complexity of this event. The range of criteria we used may not always be met for each CT saturation event. For example, the A/D clipping waveform of Figure 5 appears to contain CT saturation based on the characteristic “kneeing” in the first two cycles of the fault. However, DC offset is not present, and it was unlikely that the rating of the CT was exceeded, indicating that this event could be incorrectly classified. In addition, for greater simplicity, a CT ratio of 1200:5 was used for each event type for most of the testing irrespective of the actual CT ratio. Precise CT ratios from COMTRADE configuration files will need to be used when these tools are implemented in a production environment. If the exact CT ratio is known, then the CT’s rated current is known as well, allowing the automated process to accurately determine whether this rating was exceeded.

### 3.2. Results: Analog-to-Digital Converter Clipping

The accuracy in detecting the A/D converter clipping event was very high, achieving an accuracy of 99.27%, i.e., correctly identifying 953 out of 960 total signals processed. The threshold for the number of consecutive repeated samples was set to four samples. There were a number of events where clipping appears evident to the human eye but the repeated samples are slightly different. Those results were counted as incorrect, even though the automated process functioned properly. Utility personnel could decide whether events such as these represent a problem with the A/D converter. The A/D clipping detection methods should return proper results 100% of the time if the repeated samples have the same value; if they do not, then a minimal tolerance (e.g., 10 V or 1 A) could be allowed between the magnitudes of samples that appear to have the same value.

### 3.3. Results: Induced Transient Noise from Switching

Initial identification performance for this event was poor, at roughly 70%. To improve automated identification of induced transient noise from switching events, the automated process was modified by incorporating a rule in which the presence of ferroresonance was checked first, then harmonic resonance, and finally induced transient noise from switching, thereby ensuring that the three events do not take place at the same time. This is due to similarity of this event with others and its lack of distinguishing characteristics. In addition, a change was made to use the first cycle as a reference when isolating the disturbance, as described in Section 2.1.5. These changes resulted in an improved accuracy of 99.38%, i.e., correctly identifying 477 out of 480 total signals processed.

### 3.4. Results: High-Speed Reclosing with Tapped Motor Loads

The accuracy of this event was 100% in the tests that were conducted. However, there were only two events where the voltage did not sufficiently decay before reclosing. These events do not very often occur when utilities know the unique recloser settings of lines with tapped motor loads. Thus, a larger data set is needed to determine the accuracy of this algorithm.

### 3.5. Results: DC Offset

The DC offset algorithm is well-suited for rule-based analytics, as shown by its 99.58% accuracy, i.e., correctly identifying 956 out of 960 total signals processed. The frequency analysis method combined with the cycle mean method can accurately identify DC offset. A few signals were falsely classified as DC offset. Signals such as the CT saturation example in Figure 4 contain a steep spike at the fault inception, meaning that a DC offset may be seen in the first half-cycle. Further logic could be added in future work to account for these faults to ensure that DC offset is not detected in the first half-cycle.

### 3.6. Results: Motor Starting

Motor starting events were very straightforward to identify, with 160 out of 160 total signals being correctly identified. One reason for this 100% accuracy is that the other events we analyzed did not have many similarities with motor starting. Although transformer inrush can produce a similar signal signature, there is a differentiating factor in that motor starting is not as rich in harmonics. Motor inrush differs from single-phase faults (the most frequently occurring fault type) in that the elevated current always occurs across all three phases. For these reasons, the motor inrush classification process should be one of the most robust.

### 3.7. Results: Variable Frequency Drive Motor Starting

This event type also produced a 100% accuracy when tested, correctly identifying 160 out of 160 total signals processed. However, the ten VFD starting events used for testing were all from the same motor on the transmission system, as these devices are not extremely common. More data are needed to test the accuracy of the process for this event type.

### 3.8. Results: Melted Fuse

The accuracy in classifying melted fuse events was 99.38%, with 159 out of 159 total signals correctly identified. Melted fuse events are relatively straightforward to identify due to their short duration. One incorrect classification stemmed from an event containing a minor fault incorrectly labeled as a fuse fault. Although the fault lasted several cycles, the part of the current that exceeded the threshold was short enough to be classified as a blown fuse. Finding the fault inception and clearing points is very nuanced and may not always be 100% accurate in determining the clearing time, especially for faults that do not greatly exceed the predefined threshold (e.g., two times the rated current).

### 3.9. Results: Ferroresonance

Ferroresonance is a unique event that was classified with 99.17% accuracy by these analytics, i.e., correctly identifying 476 out of 480 total signals processed. In most of the studied data, the signals contain large gaps between samples, i.e., at least 50% of nominal peak value. A few signals did not have such large gaps, possibly due to the ferroresonance being less severe. These events were not identified as ferroresonance; new methods will need to be developed to identify these events. One such method could involve incorporating breaker statuses (i.e., open or closed) into the process, as ferroresonance usually occurs with the breaker(s) in the open state.

### 3.10. Results: Capacitor Switching

The capacitor switching classification process correctly identified 159 out of 160 total signals, resulting in an accuracy of 99.38%. The methods employed for this event type are very detailed, and are much more likely to generate false negatives than false positives. As long as the characteristic three points on the signal of Figure 16 are present, the results should be accurate. The only missed capacitor-switching event was when the voltage transient occurred on the first cycle. In this case, the rest of the processing becomes incorrect, because the nominal peak value is taken using the first cycle as a reference. This issue could be solved by using a predefined nominal peak value from an external data source for each voltage level.

### 3.11. Results: Lightning

The automated process correctly identified whether or not lightning was present for 477 out of 480 signals, for an accuracy of 99.38%. Initially, many capacitor-switching events were characterized as lightning. To remedy this, the process was updated to ensure that the presence of lightning would only be checked for if the capacitor-switching check returned negative. The lightning detection process relies on accurate determination of the duration of the disturbance. A short disturbance distinguishes lightning from other events. A few events were discovered in which the algorithm determined the disturbance to be longer than it was, which could be due to an outside disturbance unrelated to lightning. This phenomenon resulted in a few misclassifications.

### 3.12. Results: Harmonic Resonance

Harmonic resonance is difficult to distinguish from ferroresonance; thus, a modification was made to only run the harmonic resonance algorithm if ferroresonance was determined to have not occurred. This resulted in an accuracy of 100%, with 480 out of 480 signals correctly identified. There were only five different harmonic resonance event records in the dataset, for a total of fifteen voltage signals; thus, more data are needed to test the robustness of this algorithm. One future improvement that could be made is to detect resonance under the fifth harmonic, as resonance conditions can sometimes develop at these frequencies.

### 3.13. Results: Voltage Transformer Secondary Grounding

Classification for this event was very successful, with an accuracy of 99.38% on the 160 signals studied. There were many events involving improper VT secondary grounding. Many of the CT saturation fault events were not exactly 120° apart in their voltage phase angles, which would indicate improper grounding. Rule-based techniques can straightforwardly classify this event. The only issues that may occur involve inaccurate data being fed into the automated process.

### 3.14. Results: Incipient Capacitive Voltage Transformer Failure

The results for this event were not as accurate as the others, with 154 out of 160 signals (i.e., 96.25%) correctly classified as demonstrating incipient CVT failure. This lower accuracy is due to the inconsistency of CVT failure events. CVTs can be in different stages of incipient failure, and the signal signatures in these cases will not look the same. The differentiating factor is that these events are assumed to only occur one phase at a time, which improves the results.

### 3.15. Results: Cyclic Histogram-Based Continuous Signal Analysis

**Figure 21:**
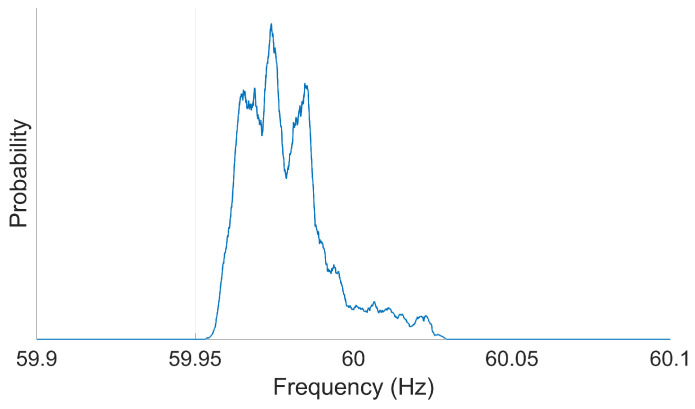
Frequency histogram for one hour of operational data from a 161 kV transformer DFR. The frequency histogram shows that the power system operated at 0.03 Hz below the intended 60 Hz without incurring a fault.

For continuous signal analysis, the developed program successfully generated the time- and frequency-based cyclic histograms and associated residual histograms from a DFR-generated CSV file containing a twenty-four-hour period of continuously recorded signals, which included all three voltage and current signals. After continuous signal processing, the required storage space was reduced by a factor of 320, i.e., 35 GB to 72 MB. Figure 3a,b provides representative examples of the time-based and residual cyclic histograms for one hour, respectively. The same hour of continuous data associated with the cyclic histogram in Figure 3a was used to generate the frequency-based histogram in Figure 21. Our current efforts are focused on integrating the developed program into a power utility’s DFR. Part of this integration involves reducing the amount of DFR compute and memory resources needed to generate the frequency-based histogram and its residual representation. The overarching goal is to use the cyclic histograms to detect deviations within the corresponding signal that would not ordinarily result in an electrical disturbance event for use in incipient prediction, detection, identification, and analysis. Ongoing work is focused on determining the best method of presenting the cyclic histograms to ensure that they are as informative as possible for PQ engineers.

## 4. Conclusions and Future Work

This work presents an approach for the automated identification of electrical disturbances within a power system. Fourteen different disturbance event types were successfully classified with an average accuracy of 99.13%, and continuous waveform data were processed and stored using a technique known as cyclic histogram, resulting in the file storage size being reduced by a factor of 320. The developed processes were validated using data collected within an operational power transmission system, and can save utility personnel time while increasing their awareness of disturbances occurring in the power system. The process presented in this paper can categorize events in minutes rather than hours or days, thereby providing utility engineers, operators, and managers with actionable intelligence to enable immediate and decisive corrective action. Impending or incipient device failures can be detected as well, allowing remedial action to be taken before complete failure to ensure the removal of safety hazards. This work can serve to increase the overall reliability of transmission systems. Lastly, this automated approach leverages institutional knowledge to create a general, simple, transparent, and explainable framework. Such transparency and explainability are lacking in universal recognition systems built on AI. This latter point is important when considering future power utility integration and adoption, as utility personnel want to know how any automated process reaches its final decision(s).

Our future work aims to increase the number of disturbance event types that can be classified and to test the process further using more data. Additional work involves investigating the scalability of the proposed approach as multiple data sources are integrated, along with its applicability to different power transmission systems. The latter will allow further assessment of the empirical thresholds used and the ease of adjusting them in order to maintain or improve classification accuracy. Future work should investigate the development and feasibility of a universal (i.e., singular) electrical disturbance identification system that removes the need for individual thresholds and tailored event identification approaches.

For the continuous waveform analysis portion, future work involves optimizing the process to reduce computing hardware requirements and further developing the presentation of the data in an informative manner.

## Figures and Tables

**Table 1:** Task runtime and percent speedup when the Python code written to generate the cyclic, residual, and frequency/energy histograms was and was not accelerated using Just-in-Time (JIT) via the “Numba” library.

Process	Python (ms)	Python + Numba (ms)	Percent Speedup (%)
Cyclic	603	13	97.84%
Residual	14	12	14.29%
Frequency and Energy	14,317	1605	88.79%
Total Combined	14,934	1630	89.09%

**Table 2:** Automated electrical disturbance event identification performance results.

Event Type	# Events	# Correct	% Correct
CT Saturation	480	464	96.67%
A/D Clipping	960	953	99.27%
Induced Transient Noise	480	477	99.38%
High-Speed Reclosing	160	160	100%
DC Offset	960	956	99.58%
Motor Starting	160	160	100%
VFD Starting	160	160	100%
Blown Fuse	160	159	99.38%
Ferroresonance	480	476	99.17%
Capacitor Switching	160	159	99.38%
Lightning	480	477	99.38%
Harmonic Resonance	480	480	100%
VT Secondary Grounding	160	159	99.38%
CVT Failure	160	154	96.25%

## Data Availability

Restrictions apply to the availability of these data. Data were obtained from the Tennessee Valley Authority (TVA) and are not available for public release due to privacy and other restrictions.

## Abbreviations

The following abbreviations are used in this manuscript:

Analog-to-Digital	A/D
Artificial Intelligence	AI
Artificial Neural Network	ANN
Configuration	CFG
COMmon format for TRAnsient Data Exchange	COMTRADE
Comma-Separated Values	CSV
Convolutional Neural Network	CNN
Current Transformer	CT
Capacitive Voltage Transformer	CVT
Data Acquisition	DAQ
Deep Neural Network	DNN
Digital Fault Recorder	DFR
Discrete Fourier Transform	DFT
Fast Fourier Transform	FFT
Gigabytes	GB
Graph Convolutional Neural Network	G-CNN
Hertz	Hz
Invertible Neural Network	INN
Just-In-Time	JIT
Kullback–Leibler	KL
Long Short-Term Memory	LSTM
Megabytes	MB
Oscillography	OSG
Process BandWidth	PBW
Power Quality	PQ
Random Access Memory	RAM
Root Mean Square	RMS
S Transform	ST
Short-Time Fourier Transform	STFT
Total Harmonic Distortion	THD
Variable Frequency Drives	VFDs
Voltage Transformer	VT
Wavelet Transform	WT
